# Annotations

**Published:** 1908-07-18

**Authors:** 


### ANNOTATIONS.

### The B.M.A. Meeting.

The seventy-sixth annual general meeting of the British Medical Association is to be held in Sheffield on July 24 at 3 p.m., under the presidency of Dr. Henry Davy. The representative meeting immediately follows this, and its agenda of business is a long one. Besides the recommendations of the Organisation Committee with regard to alterations in the standing orders, there are also put down for discussion four amendments suggested by various branches. Then follows an important special report on the proposed charter, and on the ordinances and by-laws submitted by the Council. To the latter there are no fewer than 16 amendments for consideration. After all this come the reports of the various committees, with proposed amendments of existing by-laws, and notices affecting the administration or work of the Association, and, finally, the election of committees for the ensuing year. The scientific business of the meeting will be conducted in 17 sections, which will meet on July 29, 30, and 31. The address in medicine will be delivered by Dr. Kingston Fowler, that on surgery by Mr. Pye-Smith, and the popular lecture on "Dust and Disease" by Mr. Edmund Owen. The programmes of the various sections, as usual, are full of suggestive topics which should prove fertile sources of useful discussion. Among many others of interest may be noted that of "Teething and Its Alleged Troubles," set for debate in the dental section, not that of children's diseases; a paper to be read by Dr. Wynter on a new specific for chorea; and a very full list of subjects for discussion in the electrical and army and navy sections. Compared with these last two sections the arrangements in medicine look very skimpy, but the surgeons will be hard put to transact all their business, for the list of papers is a long one, and there are to be set debates on malignant disease of the breast and on the indications for nephrotomy and nephrectomy. The only important specialty which is unrepresented seems to be that of the anæsthetists; but the multiplication of special sections is becoming so great that it is probably wise to limit the number as far as practicable. We cordially wish the Association and its members a profitable and pleasurable meeting.

### Headache Powders.

If any additional illustration were needed of the reality of the evils, recently described in these columns, of the enormous sales of "Secret Remedies and Branded Products," it is supplied by the evidence given on July 13 at Clapham during the course of an inquest held by Mr. Wyatt. The subject of this inquiry was an engine-driver, who having a headache, consumed a "Daisy headache powder," supplied to him by a local oil-shop. After taking this powder his symptoms were temporarily relieved, but not long afterwards he died somewhat suddenly. Medical evidence went to show that the deceased was suffering from influenza and rheumatism, and that the branded product in question has been found by analysis to contain acetanilide in quantities which, in different samples, vary from five to ten grains. The jury returned a verdict of death from natural causes, and a rider to the effect that in their opinion the sale of "Daisy" powders should not be allowed to continue unrestricted. As an expression of their personal sentiments this rider leaves little to be desired, but from the experience of the past it is fairly certain that its practical result will be precisely nothing. Time after time have coroners' juries made such recommendations, which are duly forwarded to the Privy Council; time after time has this very drug been made the subject of similar remarks, and on at least one of the occasions when that august body has taken the matter into consideration have the proprietors of this very product led a fierce and successful opposition. Until the *élite* of our lawgivers can be educated into a true conception of the value of health, and of laws devised to promote it, it does seem futile to attempt the much more difficult task of educating the public in the dangers of these patent medicines.

### The Sleeping Sicknesss Controversy.

The brilliant pages of the history of the advancement of tropical medicine during the last decade or two are inscribed with more than one record of heroism and devotion on the part of investigators who have paid for their successes with their lives. All the more to be deplored, therefore, is the somewhat undignified newspaper controversy that is still going on in the columns of the leading daily paper as to the priority of the discovery of the sleeping sickness trypanosome by Dr. Castellani or Col. Sir David Bruce. The former claims to have observed the parasite in cases of sleeping sickness in November 1902, six or seven months before Col. Bruce arrived in Uganda in 1903, and on the strength of this to have received less than his share of credit in connection with the matter. On behalf of the latter it is advanced by Dr. Baker, of the Uganda Medical Service, and by Sir E. Ray Lankester, Chairman in 1903 of the Committee of the Royal Society on Tropical Diseases, that when Dr. Castellani left Entebbe, after the arrival of Col. Bruce, he still maintained his now abandoned streptococcus hypothesis as the true one; that such attention as he had then paid to the trypanosome was principally due to the discovery of that parasite in two patients by Dr. Baker; and that he has admitted forming the intention, not, as it happened, carried out, of concealing from Col. Bruce material facts in connection with the problem which they were both employed by the Royal Society to attack. Sir Ray Lankester further insinuates with no little asperity that the whole dispute has arisen through a desire to advertise unfairly the London School of Tropical Medicine. In view of the approaching jubilee of the Origin of Species, inseparably connected with the superb act of generosity by which Darwin conceded to Wallace a priority really due to himself, cannot all the parties to this most unhappy bickering draw a moral from that example? Posterity regards Neptune as the discovery of both Adams and Leverrier; and, though the cases are not quite parallel, Dr. Castellani may rest assured that he will not be forgotten while Sir David Bruce is honoured and remembered.

